# The THYCOVIT (Thyroid Surgery during COVID-19 pandemic in Italy) study: results from a nationwide, multicentric, case-controlled study

**DOI:** 10.1007/s13304-021-01051-1

**Published:** 2021-04-16

**Authors:** Fabio Medas, Gian Luca Ansaldo, Nicola Avenia, Giancarlo Basili, Marco Boniardi, Marco Bononi, Aldo Bove, Paolo Carcoforo, Andrea Casaril, Giuseppe Cavallaro, Maria Grazia Chiofalo, Giovanni Conzo, Loredana De Pasquale, Paolo Del Rio, Gianlorenzo Dionigi, Chiara Dobrinja, Giovanni Docimo, Giuseppa Graceffa, Maurizio Iacobone, Nadia Innaro, Celestino Pio Lombardi, Nicola Palestini, Francesco Pedicini, Giuliano Perigli, Angela Pezzolla, Gregorio Scerrino, Stefano Spiezia, Mario Testini, Pietro Giorgio Calò, SIUEC Collaborative Group, Giacomo Anedda, Giovanni Antonelli, Giulia Arrigoni, Benedetta Badii, Elena Bonati, Antonio Mario Bulfamante, Vincenzo Candalise, Angelo Cangiano, Gian Luigi Canu, Federico Cappellacci, Alessandra Caracciolo, Ettore Caruso, D’Amore Annamaria, Eric Casal Ide, Ambra Chiappini, Calogero Cipolla, Luciana Costigliola, Federico Cozzani, Anna Crocco, Daniele Crocetti, Nicolò De Manzini, Adele Di Gioia, Velia Di Resta, Rita Eramo, Enrico Erdas, Silvia Ferriolo, Marco Filardo, Marcello Filograna Pignatelli, Rita Gervasi, Francesco Giudici, Luca Gordini, Angela Gurrado, Harmony Impellizzeri, Marco Inama, Margherita Koleva Radica, Rita Laforgia, Serafina Lattarulo, Tommaso Loderer, Roberta Lucchini, Federico Mascioli, Rosa Marcellinaro, Rosa Menditto, Giuseppina Melfa, Michele Minuto, Claudia Misso, Chiara Offi, Giuseppina Orlando, Paolo Ossola, Costantino Pagetta, Alessandro Pasculli, Renato Patrone, Iuliana Pauna, Francesca Pennetti Pennella, Dario Pietrasanta, Antonella Pino, Vito Leonardo Pinto, Stefano Piras, Andrea Polistena, Mattia Portinari, Simona Reina, Giulia Rotolo, Giulia Russo, Emilio Scalise, Lucia Ilaria Sgaramella, Maria Grazia Sibilla, Stefano Spinelli, Domenico Spoletini, Lucia Stella Curto, Martina Tascone, Francesca Torresan, Emanuela Varaldo, Elena Viviani, Annalisa Zucca

**Affiliations:** 1 Department of Surgical Sciences, University of Cagliari, Via Università 40, 09124 Cagliari, Italy; 2 Endocrine Surgery Unit, Ospedale Policlinico San Martino, Genoa, Italy; 3 General Surgery and Surgical Specialties Unit, Medical School, S. Maria University Hospital Terni and University of Perugia, Terni, Italy; 4 General Surgery Department, Endocrine Surgery Unit, Azienda USL Toscana Nord-Ovest, Pontedera, Italy; 5 Endocrine Surgery Unit, Department of Oncological-Mininvasive Surgery, Niguarda Hospital-Milan, Milan, Italy; 6 Pietro Valdoni, Department of Surgery, Sapienza University of Rome, Rome, Italy; 7 Department of Medicine, Dentistry and Biotechnology, University ``G. D’Annunzio’’, Chieti, Italy; 8 Department of Surgery, Unit of General Surgery, University Hospital of Ferrara and University of Ferrara, Ferrara, Italy; 9 Endocrine Surgery Unit, Pederzoli Hospital, Peschiera del Garda, Verona, Italy; 10 Thyroid Surgery Unit, Int Irccs Fondazione Pascale, Napoli, Italy; 11 Division of General and Oncologic Surgery, Department of Traslational Medical Sciences, University of Campania ``Luigi Vanvitelli’’, Naples, Italy; 12 Endocrine Surgery, ASST Santi Paolo E Carlo University of Milan, Milan, Italy; 13 General Surgery Unit, Parma University Hospital, Parma, Italy; 14 Division for Minimally Invasive and Endocrine Surgery, University of Messina, Messina, Italy; 15 Department of Medicine, Surgery and Health Sciences, Azienda Sanitaria Universitaria Giuliano Isontina (ASUGI), Trieste, Italy; 16 Division of Thyroid Surgery, Department of Medical and Advanced Surgical Sciences, University of Campania ``Luigi Vanvitelli’’, Naples, Italy; 17 Department of Surgical, Oncological and Stomatological Sciences, University of Palermo, Palermo, Italy; 18 Endocrine Surgery Unit, Department of Surgery, Oncology and Gastroenterology, University Of Padova, Padova, Italy; 19 Unit of Endocrine Surgery, A.O.U. Mater Domini, Catanzaro, Italy; 20 Division of Endocrine Surgery, Department of Gastroenterologic, Endocrine-Metabolic and Nephro-Urologic Sciences, Fondazione Policlinico Universitario Agostino Gemelli IRCCS, Rome, Italy; 21 Head and Neck Oncological Surgery, Candiolo Cancer Institute, Candiolo, Italy; 22 Thyroid Endocrine Surgery, Sant’Eugenio Hospital, Rome, Italy; 23 Endocrine Surgery Unit, University of Florence, Florence, Italy; 24 Division of Videolaparoscopic Surgery, Department of Emergency and Organ Transplantation, University of Bari ``A.Moro’’, Bari, Italy; 25 Department of Surgical Oncology and Oral Sciences, Unit of General and Emergency Surgery, University of Palermo, Via L. Giuffré, 5, 90127 Palermo, Italy; 26 Endocrine & US Guided Surgery OU, Ospedale del Mare, Napoli, Italy; 27 Unit of Academic General Surgery ``V. Bonomo’’, Department of Biomedical Sciences and Human Oncology, University of Bari ``A. Moro’’, Bari, Italy

**Keywords:** Thyroidectomy, Thyroid carcinoma, COVID-19, SARS-CoV-2, Endocrine surgery

## Abstract

The outbreak of the COVID-19 pandemic has led to a disruption of surgical care. The aim of this multi-centric, retrospective study was to evaluate the impact of the pandemic on surgical activity for thyroid disease among the Italian Units of Endocrine Surgery. Three phases of the pandemic were identified based on the epidemiological situation and the public measures adopted from the Italian Government (1st phase: from 9th March to 3rd May 2020; 2nd phase: from 4th May to 14th June; 3rd phase: from 15th June to 31st). The patients operated upon during these phases were compared to those who underwent surgery during the same period of the previous year. Overall, 3892 patients from 28 Italian endocrine surgical units were included in the study, 1478 (38%) operated upon during COVID-19 pandemic, and 2414 (62%) during the corresponding period of 2019. The decrease in the number of operations was by 64.8%, 44.7% and 5.1% during the three phases of COVID-19 pandemic, compared to 2019, respectively. During the first and the second phases, the surgical activity was dedicated mainly to oncological patients. No differences in post-operative complications were noted between the two periods. Oncological activity for thyroid cancer was adequately maintained during the COVID-19 pandemic.

## Introduction

Italy has been in the frontline of the COVID-19 pandemic, caused by the coronavirus SARS-CoV-2, since it exploded in the late February, and was the first among the European countries to impose a national lock-down. The outbreak of the pandemic put the National Health System under tremendous pressure, due to the need for hospitalization for a significant proportion of the infected patients. The need to devote massive resources to deal with the pandemic led to a disruption of routine surgical care, as well as activity for oncological and chronic disease.[1–5] Further, services had to adapt to reduce the risk of nosocomial infection to patients and staff, adopting new strategies of care delivery [6–9]. Initially, health authorities decided to restrict surgical activity to emergency cases, postponing elective surgical procedures of all types.

In this scenario, the surgical activity for thyroid disease too was consequently reduced. The main indications for thyroidectomy are thyroid malignancies, hyperthyroidism, and multinodular goitre with compressive symptoms. The most frequent form of thyroid neoplasm is differentiated thyroid carcinoma, characterised by a slow progression of the tumor and a good overall survival, but with a not-negligible incidence of lymph node metastases and recurrent disease, that may worsen the quality of life of the patients.[10–12]

The aim of this study was to evaluate the impact of COVID-19 pandemic on surgical activity of the Italian Units of Endocrine Surgery belonging to the SIUEC (Italian Society of Endocrine Surgery), the most important national scientific society of endocrine surgery.

For this study, we identified three phases of COVID-19 pandemic, as defined by the Italian Government based on the epidemiological situation and, consequently, the public measures adopted; the patients operated upon during these phases were compared to those who underwent surgery during the same period of the previous year. COVID-19 new confirmed cases and deaths are reported from official data released from the Italian Ministry of Health [13].

Thus, we identified:

- Phase 1 (9th March—3rd May 2020). On 9th March, the Italian government imposed a national quarantine [14]. All workplaces deemed as non-essential were closed, as well as schools and universities. Movement outside home was permitted only for emergencies or for essential workers. During this period, 201,953 COVID-19 new confirmed cases were registered in Italy, with a mean of 3 671 new daily cases, and 28 344 deaths.
- Phase 2 (4th May—14th June 2020). From 4th May 2020, manufacturing and construction sectors resumed operations, with 4.4 million workers returning to their workplaces; visits to relatives were permitted. Travel was allowed only in people’s own area of residence. Parks and public gardens reopened [15]. During this phase, there were 27,323 new confirmed cases of COVID-19, with a mean of 650 new daily cases, and 5,591 deaths.
- Phase 3 (15th June—31st August). From 15^th^ June 2020, restrictive measures were further relaxed, businesses and public spaces were gradually reopened and travels between regions were allowed [16]. During this period, 31,567 new confirmed cases of COVID-19 were registered, with a mean of 404 new daily cases, and 1,176 deaths.

## Methods

This is a multi-centric, retrospective study. We collected aggregate data from 28 Endocrine Surgical Units affiliated to the SIUEC. The inclusion criteria were: patients who had undergone thyroidectomy during the COVID-19 pandemic (from the 9th March to the 31st August 2020), defined as the case group, or during the same period of the previous year, defined as the control group.

No ethical approval from institutional board was needed for this study because only aggregate data were collected from each centre.

The objectives of the study were:

- to quantify the overall reduction of surgical activity for thyroid disease;
- to evaluate whether surgical activity for thyroid malignancies was adequately maintained;
- to verify whether the standard of care was maintained during COVID-19 pandemic, for assessing the incidence of post-operative complications;
- to investigate whether a delay in the treatment of thyroid neoplasms could have led to a higher incidence of aggressive tumors.

In pursuance of these objectives, we compared the patients who underwent thyroid surgery during COVID-19 phases with those operated upon during the same periods of the previous year.

First, a survey was conducted at each centre to assess whether the unit was within a COVID-19 hospital, as defined by each regional crisis committee, whether the ward and the personnel were reduced due to reorganisation for the COVID-19 pandemic, and whether positive cases of COVID-19 were registered among the patients or the staff.

The data collected data included for each endocrine unit:

- total number of patients operated upon for thyroid surgery
- demographic data: age, sex
- operative data: operating time, type of surgical procedure, use of Intraoperative Nerve Monitoring (IONM)
- post-operative data: post-operative stay, incidence of post-operative complications (including hypoparathyroidism, haematoma, mono-lateral or bilateral recurrent laryngeal nerve [RLN] injury)
- pathological data: benign vs malignant diagnosis; in case of malignant diagnosis: size of the main tumor, incidence of lymph node metastases, extra-thyroidal extension and multi-centricity

Re-operative thyroidectomy included both reoperations for recurrent malignant disease and completion thyroidectomy (e.g. in case of previous loboisthmectomy).

Post-surgical hypoparathyroidism was defined as PTH < 10 pg/ml, or persistent value of Calcemia < 8.00 mg/dl, or the need to administer calcium and/or vitamin D to maintain an adequate Calcemia.

RLN injury was suspected during operation due to a loss of signal at IONM (when used), or during post-operative period for dysphonia. In all these cases, a fibrolaryngoscopy was performed to assess vocal fold mobility.

According to the epidemiological phases of the COVID-19 pandemic in Italy, and to the measures adopted by the Italian government, as previously described, the patients were divided into 6 groups:

- Phase 1 (P1) group: patients operated upon from 9th March to 3rd May 2020;

- Control Phase 1 (cP1) group: patients operated upon from 9th March to 3rd May 2019;

- Phase 2 (P2) group: patients operated upon from 4th May to 14th June 2020;

- Control Phase 2 (cP2) group: patients operated upon from 4th May to 14th June 2019;

- Phase 3 (P3) group: patients operated upon from 15th June to 31st August 2020;

- Control Phase 2 (cP3) group: patients operated upon from 15th June to 31st August 2019.

Statistical analysis was performed with MedCalc® vers. 19.2.1. The Chi-squared test and Student’s t test were used for categorical and continuous variables, respectively. Results were considered statistically significant in the case of a *p *value < 0.05. Continuous variables are expressed as mean ± standard deviation of the mean.

The results of this study are reported in line with the STROCSS (Strengthening the reporting of cohort studies in surgery) criteria [17].

## Results

We enrolled in the study 3892 patients from 28 Italian surgical endocrine units, 1478 (38%) operated during Covid-19 pandemic, and 2414 (62%) during the corresponding period last year. Full results are reported in Tables 1 and 2.

**Table 1:** Demographic, surgical, pathological and post-operative data of patients treated during Covid-19 pandemic and 2019 same period time

	Phase 1 (9th March—3rd May)			Phase 2 (4th May—14th June)			Phase 3 (15th June—31st August)		
	2020	2019	*p*	2020	2019	*p*	2020	2019	*p*
Patients (*n*)	315	884		395	731		768	799	
Mean operations per unit	11.2 ± 12.3	31.6 ± 20.2	** < 0.001**	14.1 ± 10.7	26.1 ± 16.5	** < 0.001**	27.4 ± 18.9	28.5 ± 15.7	0.21
Age (year)	51.8 ± 14.3	53.6 ± 14.9	0.063	51.9 ± 14.7	55.1 ± 13.7	** < 0.001**	51 ± 15.4	53.4 ± 14.2	** < 0.001**
Gender (M/F)	83/232	202/682	0.239	103/292	202/529	0.623	232/536	218/518	0.221
Mean operating time (min)	91.7 ± 35.6	87.2 ± 31.1	**0.034**	87.3 ± 24.8	87.3 ± 32.2	1.000	91.4 ± 33.9	92.4 ± 32.2	0.549
Surgical procedure			** < 0.001**			**0.0371**			0.082
- Total thyroidectomy (*n*, %)	241 (76.5%)	679 (76.8%)		293 (74.2%)	559 (76.5%)		558 (72.7%)	619 (77%.5)	
- Loboisthmectomy (*n*, %)	44 (14%)	178 (20.1%)		83 (21%)	147 (20.1%)		165 (21.5%)	158 (19.8%)	
- Re-operative thyroidectomy (*n*, %)	30 (9.5%)	27 (3.1%)		19 (4.8%)	25 (3.4%)		45 (5.9%)	22 (2.8%)	
CLND (*n*, %)	73 (23.2%)	85 (9.6%)	** < 0.001**	22 (5.6%)	55 (7.5%)	0.264	88 (11.5%)	140 (17.5%)	** < 0.001**
LND (*n*, %)	36 (11.4%)	25 (3.1%)	** < 0.001**	9 (2.3%)	20 (2.7%)	0.79	29 (3.8%)	27 (3.4%)	0.774
Endoscopic thyroidectomy (*n*, %)	9 (2.9%)	31 (3.5%)	0.712	11 (2.8%)	28 (3.8%)	0.886	17 (2.2%)	32 (4%)	0.058
IONM (*n*, %)	134 (42.5%)	402 (45.5%)	0.404	210 (53.2%)	384 (52.5%)	0.888	456 (59.4%)	495 (62%)	0.32
Pathological diagnosis			** < 0.001**			**0.003**			0.117
- Benign (*n*, %)	109 (34.6%)	548 (62%)		235 (59.5%)	499 (68.3%)		472 (61.5%)	459 (57.4%)	
- Malignant (*n*, %)	206 (65.4%)	336 (38%)		160 (40.5%)	232 (31.7%)		296 (38.5%)	340 (42.6%)	
Post-operative stay (day)	4 ± 0.7	2.6 ± 0.9	** < 0.001**	2.5 ± 0.6	2.5 ± 0.8	1.000	2.5 ± 1.1	3.6 ± 3.4	** < 0.001**
Post-operative complications									
- Hypoparathyroidism (n, %)	57 (18.1%)	153 (17.3%)	0.818	71 (18%)	132 (18.1%)	0.962	138 (18%)	137 (17.1%)	0.717
- Haematoma (*n*, %)	5 (1.6%)	18 (2%)	0.795	5 (1.3%)	7 (1%)	0.859	7 (0.9%)	10 (1.3%)	0.684
- Monolateral RLN injury (*n*, %)	14 (4.4%)	21 (2.4%)	0.093	15 (3.8%)	15 (2.1%)	0.123	26 (3.4%)	19 (2.4%)	0.297
- Bilateral RLN injury (*n*, %)	0	0	-	0	1 (0.1%)	0.754	4 (0.5%)	1 (0.1%)	0.347

*CLND* Central compartment lymph node dissection, *LND* Lateral neck dissection, *IONM* Intraoperative Nerve Monitoring, *RLN* Recurrent Laryngeal Nerve

**Table 2:** Characteristic of the tumors treated during Covid-19 pandemic and 2019 same period time

	Phase 1 (9th March—3rd May)			Phase 2 (4th May —14th June)			Phase 3 (15th June—31st August)		
	2020	2019	*p*	2020	2019	*p*	2020	2019	*P*
Tumors (*n*)	206	336		160	232		296	340	
Maximum nodule size (mm)	19.7 ± 14.2	17.2 ± 12.6	**0.033**	15.7 ± 12.7	18.4 ± 12.9	**0.001**	18.7 ± 13.5	17 ± 12.5	0.100
Lymph node metastasis (*n*, %)	54 (26.2%)	56 (16.7%)	**0.01**	22 (13.8%)	40 (17.2%)	0.429	66 (22.3%)	68 (20%)	0.541
Extra-thyroidal extension (*n*, %)	26 (12.6%)	53 (15.8%)	0.376	22 (13.8%)	39 (16.8%)	0.496	49 (16.6%)	53 (15.6%)	0.823
Multicentric carcinoma (*n*, %)	63 (30.6%)	78 (23.2%)	0.072	50 (31.3%)	51 (22%)	0.052	82 (27.7%)	111 (32.6%)	0.205

### Characteristics of the surgical endocrine units

Eighteen (64.2%) of the 28 units included in the study were within a COVID-19 Hospital, as identified by each regional crisis committee. Following local reorganisation, 19 (67.8%) units had undergone a reduction in inpatient beds, and 14 (50%) units a reduction in personnel. In 23 (82.1%) units, COVID-19 infection was ascertained among the medical and paramedical staff, and in 15 (53.6%) units, among the hospitalised patients.

#### First pandemic phase (9th March—3rd May 2020)

During the first pandemic phase, 315 patients (P1 group) underwent thyroidectomy, with a decrease in the number of operations by 64.8% compared to 884 patients that underwent the surgery during the same period of the previous year (cP1 group). The mean patients operated per unit per period was 11.2 ± 12.3 in 2020 and 31.6 ± 20.2 in 2019 (*p* < 0.001). During this phase, a significantly longer operative time was noted in 2020, compared to 2019 (91.7 ± 35.6 min vs 87.2 ± 31.1 min; *p* = 0.034). Regarding surgical procedure, while the rate of thyroidectomy was similar among the two groups (76.5% and 76.8%), the rate of re-operative surgery was higher in the P1 group than in cP1 group (9.5% and 3.1%), whereas the rate of loboisthmectomy was lower in the first group (14% vs 20.1%; p < 0.001). Furthermore, during COVID-19 pandemic, the operations included significantly more frequently a Central Lymph Node Dissection (CLND) (23.2% of the operations in 2020 vs 9.6% in 2019; *p* < 0.001) or an Lateral Neck Dissection (LND) (11.4% in 2020 vs 3.1% in 2019; *p* < 0.001). IONM was used in about half the patients in both the groups. Post-operative stay was significantly longer in 2020 than in 2019 (4 ± 0.7 days vs 2.6 ± 0.9 days; *p* < 0.001). The incidence of post-operative complications was similar among the two groups. Indication for surgery was more frequently a malignancy during 2020, compared to 2019. In fact, pathological diagnosis revealed a malignancy in 206 (65.4%) patients of P1 group, and in 336 (38%) of cP1 group (*p* < 0.001). Further, patients in P1 group had a significantly larger nodule size (19.7 ± 14.2 mm vs 17.2 ± 12.6; *p* = 0.033) and a higher incidence of lymph node metastases (26.2% vs 16.7%; *p* = 0.01) compared to patients in cP1 group. No significant differences were revealed regarding the incidence of extra-thyroidal extension and multi-centric tumors among the two groups.

#### Second pandemic phase (4th May—14th June 2020)

During the second pandemic phase, 395 patients (P2 group) underwent thyroid surgery, vis-à-vis 731 patients during the same period of 2019 (cP2 group), marking a reduction of surgeries by 44.7%. The mean patients operated per unit per period was 14.1 ± 10.7 in 2020 and 26.1 ± 16.5 in 2019 (*p* < 0.001). Patients operated upon during the COVID-19 pandemic were younger, compared to those operated during the previous year (51.9 ± 14.7 years old vs 55.1 ± 13.7 years old; *p* < 0.001). During this phase, the operative time and the rate of central and lateral neck lymphectomy performed were similar among the two groups, whereas the surgical procedure consisted more frequently of secondary surgery in P2 group (4.8% vs 3.4% of cP2 group; *p* = 0.0371). Post-operative stay, the use of IONM and the incidence of post-operative complications were similar among the two groups. Further, in this phase, pathological diagnosis was more frequently a malignancy during COVID-19 pandemic, compared to 2019 (40.5% vs 31.7%; *p* = 0.003). However, patients in P2 group had a significantly smaller nodule size (15.7 ± 12.7 mm vs 18.4 vs 12.9 mm; *p* < 0.001); no significant differences were noted regarding the incidence of lymph node metastases, extra-thyroidal extension and multi-centric tumors among the two groups.

#### Third pandemic phase (15th June—31st August)

During the last pandemic phase, 768 patients (P3 group) underwent thyroidectomy, with a decrease in number of operations by 5.1%, compared to 799 patients in the same period of 2019 (cP3 group). The mean patients operated per unit per period was similar between the two groups (27.4 ± 18.9 vs 28.5 ± 15.7; *p* = 0.21). As in the previous phase, a younger age was noted in patients operated upon during COVID-19 pandemic (51.5 ± 15.4 vs 53.4 ± 14.2; *p* < 0.001). The mean operative time and the surgical procedures were similar among the two groups. A significantly lower rate of CLND was noted in P3 than in cP3 group (11.5% vs 17.5%; *p* < 0.001), whereas the rate of LND was similar between the two groups. IONM was used in about 60% of the patients in both the groups. Post-operative stay was shorter in P3 group than in the control group (2.5 ± 1.1 days vs 3.6 ± 3.4 days; *p* < 0.001). The incidence of post-operative complications was similar among the two groups. Pathological examination revealed a similar incidence of malignancy in the two groups (38.5% vs 42.6%; *p* = 0.117), and no significant differences were found regarding the pathological features of the tumors among the two groups.

## Discussion

**Fig. 1:**
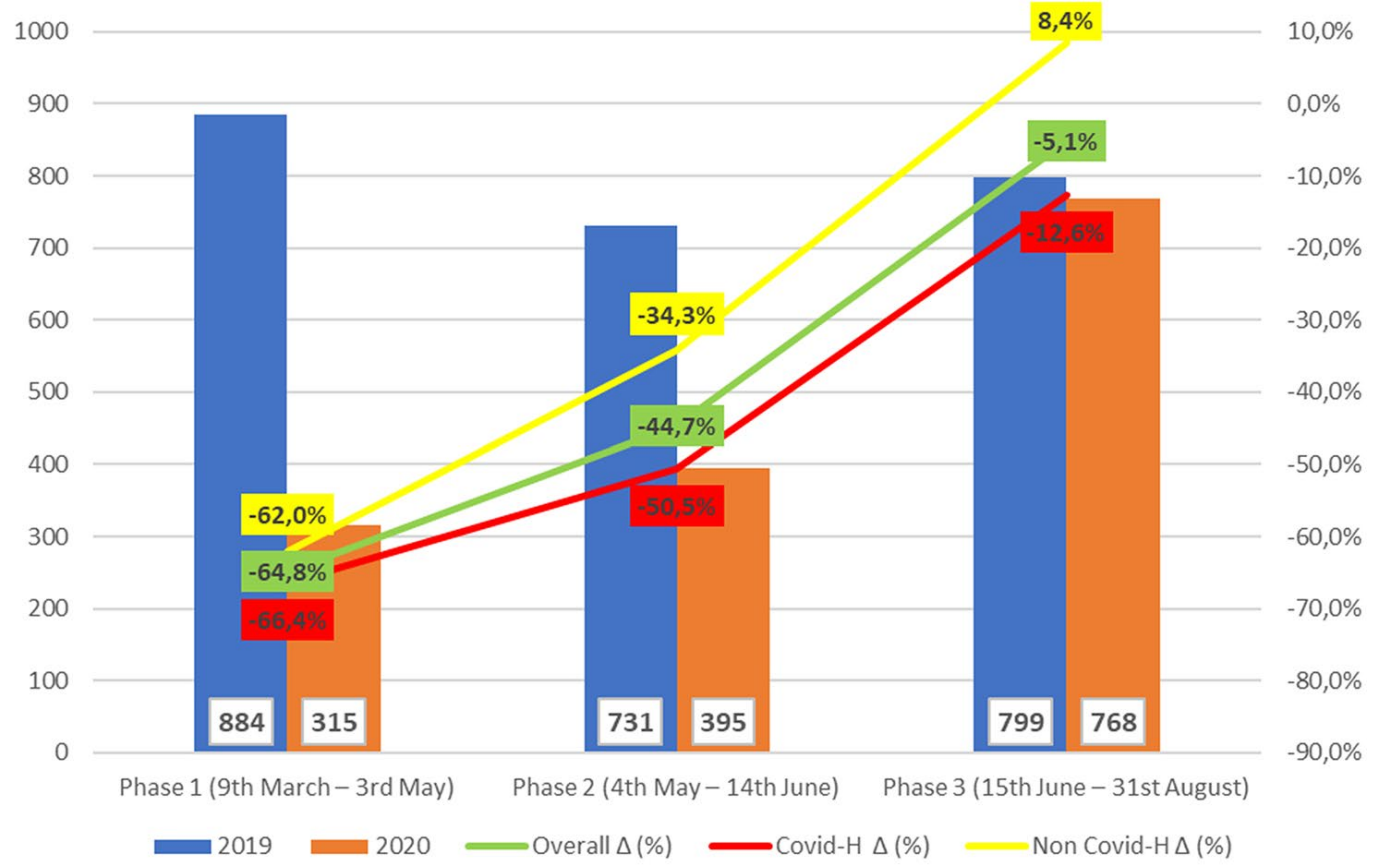
The bars indicate the number of patients operated upon during  2019 (blue bars) and  2020 (orange bars), divided according to the pandemic phase. The lines indicate the difference (∆) between patients operated in 2020 and 2019, expressed as a percentage. The green line represents the overall difference, the red line the difference reported in Covid-19 Hospitals (Covid-H), and the yellow line the difference reported in Non- Covid-19 Hospitals. Y axis: the number of patients is indicated on the left; the difference between patients operated in 2020 and 2019, expressed as a percentage, is represented on the right

Our study has ascertained and quantified the reduction in surgical activity for thyroid disease in the Italian Surgical Endocrine Units during the COVID-19 pandemic. As illustrated in Fig. 1, compared to the same periods of 2019, the reduction of operations was 64.8% during the first phase, 44.7% during the second phase, and 5.1% during the third phase. As expected, the decrease of activity was more consistent in the units belonging to COVID-19 hospitals, while was lower than in non-COVID-19 hospitals, where, indeed, an increase of operations of 8.4% was noted during the last phase, compared to 2019.

**Fig. 2:**
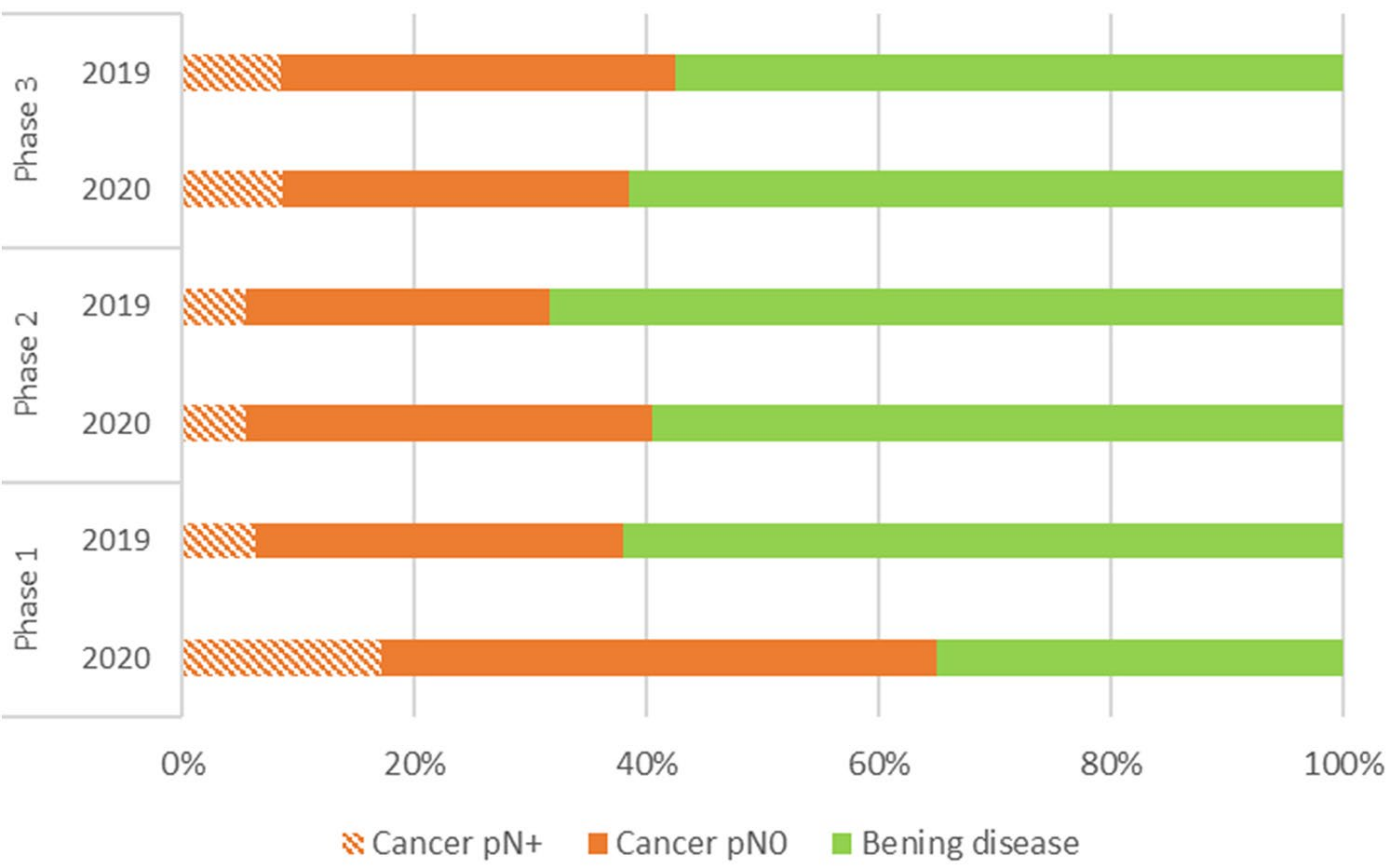
The bars indicate the pathological diagnosis of patients operated upon during 2019 and 2020, divided according to the pandemic phase, expressed as a percentage. In each bar, the part with orange diagonal stripes indicates the proportion of thyroid carcinoma with lymph node metastases (pN+), the orange part indicates the proportion of thyroid carcinoma without lymph node metastases (pN0), and the green part indicates the proportion of benign disease. Phase 1: from 9th March to 3rd May; phase 2: from 4th May to 14th June; phase 3: from 15th June to 31st August

Certainly, the first phase has been the most dramatic. Suddenly, the personnel had to deal with an almost unknown disease, and to fully reorganize the activity. During this phase, elective surgery was suspended in most of the hospitals, and only oncological and urgent operations were allowed. As evidenced from our study, only a few operations were performed in this phase; as illustrated in Fig. 2, most of the patients had a thyroid malignancy, many of which with aggressive features, and the operations consisted often of aggressive surgery, with a high rate of CLND and LND, as also demonstrated from the significantly longer operative time in this phase.

During the second phase, we witness an initial recovery of the activity. In this phase, the main indication for surgery was a thyroid malignancy, but the pathological features of the tumours were similar, compared to the same period of 2019.

The third phase represented a full recovery of the activity, allowed by the improvement of the epidemiological situation and of the organization, including the availability of rapid pre-admission tests. During this period, elective non-oncological activity was ubiquitously resumed, and many patients with benign thyroid disease were operated upon.

Overall, our data demonstrate that even in the critical conditions experienced during the COVID-19 pandemic, oncological activity for thyroid malignancies was adequately maintained, and that the most aggressive cases were treated promptly even during the first phase.

It is interesting to note that, during the COVID-19 pandemic, the operated patients were younger, compared to those who had undergone surgery during the same period of 2019. This finding could be explained by the fact that during the pandemic, the hospitals have represented clusters of infections; hence, probably, the surgeons chose younger patients, to reduce the risk of severe disease in case of a COVID-19 infection; further, it is possible that older patients decided to refuse hospitalisation for surgery, alarmed by the possibility of a nosocomial infection.

Another issue of our study was to evaluate whether the standard of care was maintained during COVID-19 pandemic.

First, we did not observe any significant differences in the use of the IONM during the COVID-19 pandemic. As is known, one of the most harmful manoeuvres for medical equipment, in the case of a patient with COVID-19 infection, is orotracheal intubation; the IONM requires a careful placement of the endotracheal tube, because the electrodes must be placed exactly between the vocal folds. The utilisation of appropriate personal protective equipment, particularly by the anaesthesiologists, has allowed an adequate utilisation of IONM, which, overall, was used in 54.1% of the operations during COVID-19 pandemic and in 53.1% during the same period of 2019.

In addition, the incidence of post-operative complications was comparable between the two periods. Post-operative hypoparathyroidism was observed in about 18% of the patients, with insignificant variations among the various phases taken into consideration, and the incidence of post-operative bleeding and of RLN injury were similar among the two periods.

It is interesting to note the variation in the post-operative stay during the three phases of the pandemic. In fact, our study revealed a longer hospitalisation during the first phase, compared to the same period of 2019. The observed difference is partly justified by a higher rate of complex procedures (reoperations for cancer recurrence and thyroidectomies with lymph node dissection). It could also be explained by the fact that hospitalisation was challenging during this phase, on the one hand due to a reduction in inpatient beds, and on the other hand, since the results of pre-admission tests, including the nasopharyngeal swab, were taking a long time. For these reasons, the surgeons probably decided to retain the patients at the hospital for a longer time, to exclude any possible post-operative complication that could have required a further hospitalisation. On the contrary, we observed a shorter post-operative stay in the third phase of the COVID-19 pandemic; during this phase, a high turnover of inpatient beds was required to fully resume surgical activity.

Finally, we evaluated whether a delay in the treatment of thyroid neoplasms could have led to a higher incidence of aggressive tumors. Overall, we observed an incidence of aggressive features comparable to the same periods of the previous year.

## Conclusion

While we are writing, unfortunately, we are witnessing a new escalation of COVID-19 pandemic. Considering the data of our article, we would urge the following for consideration:

- Surgeries should be performed maintaining the safety of healthcare staff and patients, with better planning pre-admission triage and tests, the use of personal protective equipment, and safe management of surgical smoke;
- Oncological surgery for thyroid cancer should be ensured, even in the case of new restrictions of activity;
- Surgery for patients with nodules with cytological diagnosis of follicular neoplasm or suspicious for a follicular neoplasm, corresponding to THY-IV nodule according to Bethesda classification, should not be postponed, due to the risk of tumor growth and extension;
- Screening programs and planned visits for thyroid disease, including surveillance for patients with thyroid cancer, should not be delayed. We suggest, in case of limitation in hospital access, that these activities be moved to extra-hospital settings, with proper approvals from regional and local health systems.

## Data Availability

The datasets generated during and/or analysed during the current study are available from the corresponding author on reasonable request.
